# Deep Thought on the HIV Cured Cases: Where Have We Been and What Lies Ahead?

**DOI:** 10.3390/biom15030378

**Published:** 2025-03-05

**Authors:** Qing Xiao, Sanxiu He, Chaoyu Wang, Yixing Zhou, Chensi Zeng, Jun Liu, Tingting Liu, Tingting Li, Xi Quan, Linyue Wang, Liuyue Zhai, Yi Liu, Jun Li, Xiaomei Zhang, Yao Liu

**Affiliations:** Chongqing Key Laboratory of Translational Research for Cancer Metastasis and Individualized Treatment, Department of Hematology-Oncology, Chongqing University Cancer Hospital, Chongqing 400030, China

**Keywords:** human immunodeficiency virus (HIV), functional cure, viral reservoir, allogeneic stem cell transplantation (allo-HSCT), cell therapy, gene therapy, immunotherapy

## Abstract

Antiretroviral therapy (ART) can effectively suppress the replication of human immunodeficiency virus (HIV), but it cannot completely eradicate the virus. The persistent existence of the HIV reservoir is a major obstacle in the quest for a cure. To date, there have been a total of seven cured cases of HIV worldwide. These patients all cleared HIV while undergoing allogeneic stem cell transplantation (allo-HSCT) for hematological malignancies. However, in these cases, the specific mechanism by which allo-HSCT leads to the eradication of HIV remains unclear, so it is necessary to conduct an in-depth analysis. Due to the difficulty in obtaining donors and the risks associated with transplantation, this treatment method is not applicable to all HIV patients. There is still a need to explore new treatment strategies. In recent years, emerging therapies such as neutralizing antibody immunotherapy, chimeric antigen receptor T cell (CAR-T) therapy, gene editing, and antiviral therapies targeting the reservoir have attracted wide attention due to their ability to effectively inhibit HIV replication. This article first elaborates on the nature of the HIV reservoir, then deeply explores the treatment modalities and potential success factors of HIV cured cases, and finally discusses the current novel treatment methods, hoping to provide comprehensive and feasible strategies for achieving the cure of HIV.

## 1. Introduction

Acquired immune deficiency syndrome (AIDS) is an infectious disease caused by human immunodeficiency virus (HIV) infections [1]. According to the results of the Global Burden of Disease analysis, it is estimated that in 2021, 40 million people were infected with HIV globally, and 718,000 people died from HIV-related diseases [2]. Although antiretroviral therapy (ART) can effectively reduce the HIV infection rate and mortality, it requires lifelong medication and has significant side effects [3]. Many concerns regarding ART, such as drug interactions, toxicity, cost, viral resistance, patient compliance, and non-AIDS-related morbidities, have driven the pursuit of a cure [4,5].

One of the major obstacles to curing HIV is the difficulty in targeting the HIV reservoir [6]. In latently HIV-infected cells, mostly resting CD4^+^ T cells, proviral DNA transcription is scant or absent [7]. Lacking viral protein expression, these cells dodge detection and destruction by innate and adaptive immune systems [8]. A sterilizing cure might be achieved if the latently infected cells could be eliminated and replaced by uninfected cells or, ideally, cells with inherent resistance to HIV infection through allo-HSCT [9]. This approach has been attracting the interest of researchers for many years. To date, only seven cases have been declared successfully cured worldwide [10,11,12,13,14,15,16]. In these cases, all the patients were infected with HIV and suffered from hematological malignancies simultaneously. Moreover, the HIV in their anatomical sites was eliminated when they received allo-HSCT for the treatment of hematological malignancies [17]. Among them, the transplanted cells of five cured patients had specific homozygous deletions in the CCR5 co-receptor gene, making them resistant to CCR5-tropic strains (but not CXCR4-tropic strains). Those of the other two cured patients had no such homozygous deletions and were wild-type or heterozygous. These cases suggest that allo-HSCT from suitable donors is feasible for treating both hematological malignancies and HIV [18]. Nevertheless, in allo-HSCT treatment, it is still not entirely clear which factors contribute to the cure of HIV [19].

Although the current seven cured cases of HIV were achieved through allo-HSCT, the limited donor sources and the high risks associated with transplantation have severely restricted the application of allo-HSCT in HIV treatment [20]. It is feasible to develop new treatment methods based on the elements that might have led to HIV elimination in the cured cases [21]. In recent years, emerging therapies such as neutralizing antibody immunotherapy, chimeric antigen receptor T cell (CAR-T) therapy, and gene editing have attracted extensive attention due to their effectiveness in suppressing HIV replication [22,23,24,25]. However, these treatment strategies have not yet reached the mature stage of research and development, and there is still a long way to go before achieving a cure for HIV. Summarizing the mechanism, research status, and existing deficiencies of these treatment methods will be beneficial to the advancement and implementation of subsequent research work.

In summary, the current situation of HIV treatment is moving towards greater efficiency, accessibility, and closer to a cure. Although challenges remain, ongoing innovations and research offer new hopes and possibilities for people living with HIV (PLWH). This review summarizes the latest advances in sterilizing and functional cures for HIV infection in recent years to explore directions for future research.

## 2. The HIV Reservoir

The HIV reservoir refers to the infected cells that have integrated complete HIV DNA, stably exist over a long period under ART, and whose viral genomes are capable of infecting new cells [26]. It mainly consists of resting CD4^+^ T cells, macrophages, dendritic cells, etc. [27,28]. These cells can be found not only in the peripheral blood but also distributed in lymphoid tissues and anatomical locations that are hard for drugs to reach, such as the gut mucosa-associated lymphoid tissue and the central nervous system [29,30]. The persistent HIV reservoir is the primary factor leading to viral rebound after the discontinuation of ART, and it also poses the main challenge in the current treatment of HIV infection [6]. The description of the formation mechanism, duration, and immune characteristics of the reservoir helps to gain a more explicit understanding of the reasons why the HIV reservoir is so difficult to eradicate.

### 2.1. Formation Mechanism

In HIV infection, one of the main cell types of the reservoir is generally considered to be resting CD4^+^ T cells [8,31,32,33]. The general view of the origins of latently infected resting memory CD4^+^ T cells is that they arise from infection of CD4^+^ T cells activated during an immune response as the activated T cells return to an immunologically resting state [34,35]. However, the latest research shows that HIV can directly infect resting T cells to form latently infected cells. Moreover, productively infected cells also originate from these directly infected resting T cells [36]. Besides resting CD4^+^ T cells, macrophages and dendritic cells are also important reservoirs for HIV. These cells are affected by specific states, such as the compact structure of cellular chromosomes, the limitation of transcription factors (such as p-TEFb, NF-κB), and the obstruction of the post-transcriptional transport of RNA out of the nucleus, which leads to the virus being in a dormant state [6,37,38,39].

### 2.2. Reservoir Duration

To eradicate the HIV reservoir, it is essential to figure out when the HIV reservoir is established. Some research has attempted to achieve HIV remission through ART initiation in the early stage of HIV infection. However, in most cases, even when remission is initially achieved, it eventually leads to viral rebound [40,41]. An animal model study found that rhesus monkeys started receiving a 24-week ART within 3 days after being artificially infected with simian immunodeficiency virus (SIV). After the treatment was interrupted, the median time to viral rebound was 21 days, indicating that the HIV reservoir was initially established in the early stage of infection [42,43]. Although ART in the early stage of infection cannot eradicate HIV, starting ART as early as possible does help reduce the size of the viral reservoir [44,45]. Theoretically, under the effect of continuous ART, HIV will be gradually eliminated as the infected cells die. However, the existence of long-lived infected cells (such as resting T cells) makes the eradication of HIV a distant prospect. In infected cells, HIV DNA exists in two forms: free DNA and integrated proviral DNA (the main form). The half-life of free DNA is very short (about 2.9 days), while the half-life of integrated proviral DNA is approximately 44 months [46,47]. That is to say, if relying solely on ART, it would take 73 years for the reservoir to naturally decay to zero, which is also the reason why PLWH currently need to take medication for life [47]. It can be seen that the HIV reservoir has the characteristics of being established early and having a long half-life. 

### 2.3. Immune Characteristics

Under the effect of ART, the quantity and function of immune cells in 60–90% of PLWH can be restored to a certain extent [48]. But why is it that the reconstituted immune system still fails to eradicate HIV? This is closely related to the immune characteristics of the latent HIV reservoir, mainly in the following three aspects: ① The latent viruses in the reservoir are predominantly in a dormant state, hardly expressing complete viral proteins, and the infected cells lack obvious immune markers [49,50]. The immune system mainly targets active viruses and has limited ability to recognize latent viruses. ② The HIV reservoir is mainly concentrated in cell populations that highly express immune checkpoint molecules such as PD-1, CTLA-4, TIGIT, LAG-3, TIM-3, and CD160, and these cell populations can escape immune surveillance [51,52,53]. ③ The “dormant” HIV reservoir is not static. Latent reservoir cells may occasionally divide even though they are predominantly in the resting state. This clonal expansion allows the reservoir to actually sometimes grow as well as decline despite being on ART [54,55,56]. Current antiretroviral therapies do not target HIV transcription or translation; therefore, spontaneous viral gene transcription and translation can persist during ART [57]. Most of the integrated proviral DNA is defective [58,59,60], so the spontaneously “active” reservoir cells predominantly exhibit abortive transcription. However, these products of abortive transcription and translation can still maintain HIV-specific CD4^+^ and CD8^+^ T cell responses during suppressive ART [57]. Ongoing expression of viral genes leads to the immune exhaustion of HIV-specific T cells [61,62,63]. All in all, the HIV reservoir has unique immune characteristics. It can both escape immune surveillance and cause immune exhaustion. Relying solely on the host’s own immunity may not be sufficient to deplete the viral reservoir.

To sum up, the major reasons that prevent the eradication of the viral reservoir are as follows: ① Some reservoirs are in drug-inaccessible anatomical sites; ② the reservoir is established at an early stage; ③ the reservoir cells have a long lifespan; ④ the half-life of proviral DNA is long; ⑤ the reservoir lacks unique biomarkers; ⑥ some reservoir cells highly express immune checkpoint molecules; and ⑦ the reservoir continuously exhausts the immune system. Although there are many strategies for clearing the reservoir, only allo-HSCT can truly overcome these issues to eradicate HIV. Developing a universally applicable treatment for all PLWH remains difficult. It is worth noting that, apart from the challenges posed by the inherent characteristics of the HIV reservoir itself, the current scarcity of various technologies aimed at quantitatively analyzing and continuously monitoring the HIV reservoir is also one of the major obstacles to HIV cure [64]. Since many excellent reviews have comprehensively elaborated on this field previously [49,65,66], this article will not go into detail.

## 3. The Definitions of HIV Cure

In HIV cure research, what does “cure” mean? The International AIDS Society (IAS) classifies the situations of HIV cures into two categories: sterilizing HIV cure and functional HIV cure [67]. Sterilizing HIV cure means the elimination of all HIV-infected cells; functional HIV cure refers to the lifelong control of the virus without ART [68]. In patients with functional HIV cure, the HIV viral load is undetectable, but this does not mean the complete eradication of all HIV in patients’ anatomical sites [69]. In this state, the patient’s immune system can naturally recognize and eliminate infected cells, control free viruses and actively replicate viruses, and prevent HIV from reactivating and causing clinical symptoms [70]. In contrast, achieving a sterilizing HIV cure requires the complete elimination of all infected cells and viruses, including the latent viruses hidden within the immune system and tissues [71]. The determination of HIV cure can be defined by specific biomedical indicators after the discontinuation of ART: ① undetectable viral load; ② no loss of CD4^+^ T cells; ③ no disease progression; and ④ no risk of HIV transmission [67]. Among the cases declared cured, although multiple test results indicate the elimination of HIV, trace amounts of HIV-1 DNA and 2-LTR circles can still be transiently detected during the analytical treatment interruption (ATI) period [10,11,12,13]. The clinical significance of this test result remains unclear, and further follow-up is needed to understand their impact. Therefore, it is still difficult to determine whether these cured cases have achieved sterilizing HIV cure, but at least they have met the criteria for a functional cure.

There remain two aspects that have not been clearly defined in the assessment criteria for HIV cure. Firstly, the duration of an undetectable viral load, and secondly, the anatomical subject for HIV detection. Among the seven cured cases, the shortest time from the suspension of ART to the announcement of long-term remission was less than two years (“Berlin patient”), while the longest was six years (“The second Berlin patient”) [10,16]. Several other cases of allo-HSCT in PLWH have, however, failed to cure HIV-1 infection [17,72]. For these patients, HIV could be re-detected within ten months after the interruption of ART. Among non-allo-HSCT treatment failure cases, the Mississippi baby had a relatively late viral rebound. She stayed HIV-negative for 27 months after stopping ART and was once thought to be functionally cured. But in July 2014, HIV was detected in her body again [40]. Thus, an observation period of at least 30 months after stopping ART is a convincing time point for determining a functional cure; research indicates that after allo-HSCT, the virus is cleared in a tissue-specific and step-by-step manner [73,74]. HIV and the infected cells are first cleared from the peripheral blood, then from the peripheral lymph nodes, and finally from the mesenteric lymph nodes that drain the gastrointestinal tract. Therefore, clearing the viral reservoir in the blood is the first benchmark for HIV cure after allo-HSCT. However, the complete eradication of the viral reservoir still needs to be confirmed by analyzing the mesenteric lymph node compartment [17]. Given the difficulty in testing the mesenteric lymph node compartment, anti-HIV immunity can be used as a surrogate indicator for the presence or absence of residual virus. A recent study has demonstrated that the decline rate of anti-HIV antibody levels is slower than that of the viral reservoir, so the attenuation of anti-HIV T cell immunity can also be used as evidence for the elimination of the viral reservoir [75].

## 4. Cases of Sterilizing HIV Cured by Allo-HSCT

### 4.1. HIV Cure Cases

The emergence of ART has brought hope to PLWH, significantly prolonging their survival time, yet it fails to eradicate the virus [76]. Since the first cured case was reported in 2008, new cured cases have emerged one after another, and there have been a total of seven cases so far [77]. Although all of these seven cases were cured through allo-HSCT, there are differences in aspects such as anti-cancer regimens, pre-transplant virus control, pre-transplant conditioning, donor sources, post-transplant integration, graft-versus-host disease (GVHD), and ART stop time [19]. In order to optimize the allo-HSCT treatment regimens and explore the potential mechanisms of HIV cure, this article conducts an in-depth analysis of these seven cured cases from these aspects (Table 1).

#### 4.1.1. Berlin Patient

The “Berlin patient” was the world’s first case to be officially declared as “cured of HIV” [10]. In 1995, this patient was diagnosed with HIV infection (predominantly R5-tropic, with X4-tropic) [78]. In 2007, he was hospitalized due to acute myeloid leukemia (AML). For four years prior to admission, he had undergone ART treatment. Upon admission, his CD4^+^ T cell count was 415 per cubic millimeter and no HIV RNA was detected. The initial treatment for AML comprised two rounds of induction chemotherapy and one round of consolidation chemotherapy. Seven months after presentation, AML relapsed, and the patient underwent allo-HSCT with CD34^+^ peripheral blood stem cells from an HLA-identical donor who had been screened for homozygosity for the CCR5 delta32 allele (CCR5 Δ32/Δ32). The CCR5 Δ32/Δ32 mutation involves a 32-base pair deletion in the CCR5 gene, resulting in a nonfunctional receptor [79]. This genetic trait is significant because the CCR5 receptor is one of the main entry points for exclusively CCR5-tropic HIV to infect cells. Therefore, individuals with the CCR5 Δ32/Δ32 mutation are highly resistant to HIV infection. The patient had been continuously receiving ART until the day before the transplantation, and complete chimerism of the graft was achieved 13 days after the transplantation. Grade I graft-versus-host skin disease was treated with cyclosporine after transplantation. A second transplantation from the same donor took place 391 days after the first, preceded by a single dose of total body irradiation (200 cGy). Until his death from cancer in 2020, the virus level in his body remained undetectable [80,81].

#### 4.1.2. London Patient

The London patient was diagnosed with HIV infection in 2003 and stage IVB Hodgkin lymphoma (HL) in December 2012 [11,82]. He received ART while undergoing chemotherapy and salvage treatment. Drug resistance occurred, resulting in a switch to ART, and this regimen controlled the HIV reservoir at a level below 0.286 infectious units per million T cells. Later, the patient received allo-HSCT. The donor cells had an allele mismatch at HLA-B and carried the homozygous mutation of the CCR5 gene (CCR5 Δ32/Δ32). A conditioning regimen was administered before transplantation, and anti-CD52 (Alemtuzumab), cyclosporine A, and methotrexate were used after transplantation to prevent GVHD. Complete donor chimerism was achieved 30 days after transplantation. The patient developed a fever and grade I gastrointestinal GVHD on day 77, which resolved without intervention. His ART regimen was maintained after allo-HSCT and interruption was started on day 510. Thirty months after the discontinuation of HIV treatment, the patient’s HIV viral load remained below the detectable limit, and he was declared “cured of HIV”.

Similar to the “Berlin patient”, the “London patient” also underwent allogeneic transplantation with CCR5 Δ32/Δ32, and a certain degree of GVHD in T cells was also observed after the transplantation. The difference is that the “Berlin patient” was a heterozygote for CCR5 Δ32/WT, received total body irradiation and intensive conditioning during both allo-HSCTs and immediately stopped ART during the first allo-HSCT. In contrast, the “London patient”, as an individual with CCR5 WT/WT, only received mild conditioning and a single allo-HSCT, without total body irradiation or interruption of ART during the allo-HSCT, yet was also able to achieve long-term remission.

#### 4.1.3. Dusseldorf Patient

The third cured patient was diagnosed with HIV (mainly R5-tropic, with an extremely low proportion of X4-tropic) in January 2008 [12]. The patient received ART in October 2010, and his plasma viral load was continuously suppressed. In January 2011, the patient was diagnosed with AML and had two relapses after achieving cancer remission through chemotherapy. Before undergoing allo-HSCT using CCR5 Δ32/Δ32 donor cells (with 10/10 HLA matching) in February 2013, he received reduced-intensity conditioning (RIC). Thirty-four days after allo-HSCT, complete donor chimerism was established and maintained. Although cyclosporine, mycophenolate mofetil, and tacrolimus were given as immunosuppressive therapies, he still developed persistent mild chronic ocular GVHD and bilateral keratoconjunctivitis sicca. ART was continued throughout the treatment process and discontinued 69 months after allo-HSCT. Since then, he has remained in HIV remission. Four years after ATI, there was no viral rebound and a lack of immunological correlation regarding the persistence of HIV-1 antigens.

The “Dusseldorf patient” was a heterozygote for CCR5 Δ32/WT. Like the previous two cured patients, he also achieved long-term remission after CCR5 Δ32/Δ32 allo-HSCT and mainly carried the R5-tropic virus strain. The main difference is that the stem cells transplanted into the Dusseldorf patient were sourced from a donor of the opposite sex, which indicates that donor-recipient gender mismatch does not affect the outcome of HIV cure.

#### 4.1.4. New York Patient

The New York patient was diagnosed with acute HIV (R5-tropic, without X4-tropic HIV-1) infection four years prior to the diagnosis of acute myeloid leukemia AML [13]. Her antiviral treatment regimens had changed several times, yet the viral level had always been controlled below 30 HIV-1 RNA copies/mL. During the induction chemotherapy period, her transient low-level viremia reached 150 HIV-1 RNA copies/mL. In 2017, she underwent a haplo-cord transplant consisting of CD34-selected cells from the peripheral blood of the haploidentical adult donor, followed by the 5/8 HLA-matched CCR5Δ32/Δ32 CBU. Fludarabine, melphalan, total body irradiation, and anti-thymocyte globulin were used as transplant conditioning regimens. Mycophenolate mofetil and tacrolimus were administered for prophylaxis against GvHD. By the 14th week, complete chimerism occurred in both lymphoid and bone marrow-derived cells. Throughout the post-transplant period, she remained aviremic for HIV during intermittent ART and did not develop acute or chronic GVHD. ART was interrupted 37 months after the transplant, and no HIV RNA was detected within 18 months after ATI, indicating HIV-1 remission and a possible HIV-1 cure.

Compared with the previous three cured cases, this cured case has many differences: ① The “New York patient”, a mixed-race female, is the sole reported case achieving long-term HIV remission post-haplo-cord transplant. Her odds of finding a fit donor were minuscule. Using CCR5 Δ32/Δ32 cord blood cells eased matching needs and unlocked new prospects for future allo-HSCT treatment of PLWH. ② This patient did not develop GvHD. In contrast, GvHD was crucial for the previous three HIV cures. This also shows that the risks of graft rejection and GvHD in cord blood stem cell transplantation are likely lower than those from other sources like peripheral blood or bone marrow. ③ The “New York patient” completely lost the HIV-specific antibody response throughout the post-transplant period, which is a unique feature. While the “Berlin patient”, “London patient”, and “Düsseldorf patient” did not exhibit a completely negative HIV antibody response, it did gradually diminish over time. ④ The CCR5 Δ32/Δ32 cells of the “London patient” and “Düsseldorf patient” were susceptible to CXCR4-tropic strains, while those of the “New York patient” showed resistance to both R5- and X4-tropic strains [13]. Given the high CXCR4 in CB cells, this is astonishing and was probably due to different in vivo stem cell differentiation [13].

#### 4.1.5. City of Hope Patient

The “City of Hope patient” had been infected with HIV (predominantly R5-tropic) for 31 years before being diagnosed with AML [14]. He had a medical history of undetectable HIV RNA levels during ART, and his AML went into remission after salvage chemotherapy. The patient was a homozygous recipient with wild-type CCR5, but the donor of the hematopoietic stem cells carried the CCR5Δ32/Δ32 mutation. Allo-HSCT was carried out using RIC with fludarabine and melphalan, along with sirolimus–tacrolimus for prophylaxis against GVHD. The patient experienced chronic oral GVHD (grade I) 17 months after allo-HSCT. Twenty-five months after allo-HSCT, ART was halted, and he stayed HIV-free for 35 months after the ART cessation.

Among all the mentioned cured cases, the “City of Hope patient” was the eldest and had been infected with HIV for the longest time. Compared with other cure cases, this patient underwent an RIC protocol (fludarabine and melphalan) for conditioning and received less intensive immunosuppressive prophylaxis (tacrolimus and sirolimus) against GVHD, aiming to make the treatment more bearable for the elderly. This case indicates that for older adults with long-term HIV infection, there remains a chance of attaining long-term remission and a cure.

#### 4.1.6. Geneva Patient

The sixth reported long-term HIV remission case is the “Geneva patient” [15]. He was diagnosed with HIV-1 in 1990 and started ART right away. But after 9 years of treatment, full viral suppression was not achieved. In 2005, a lopinavir-based treatment got the plasma viral load under control. In January 2018, he was diagnosed with myeloid sarcoma that affected the lymph nodes and bone marrow. After two cycles of induction chemotherapy, he experienced a short-term relapse and then received hypomethylating agent treatment. Subsequently, he underwent allo-HSCT (CCR5 WT/WT) in July 2018. The donor was an unrelated male with a nine-of-ten HLA match and without the CCR5 Δ32 mutation. Before the transplantation of peripheral stem cells (without T cell depletion), he received a cycle of sequential conditioning regimen. Cyclophosphamide, tacrolimus, and mycophenolate mofetil were used to prevent GvHD. Less than a month after the transplantation, complete donor chimerism was achieved. This individual successively developed acute hepatic GvHD, mild chronic cutaneous GvHD, recurrence of hepatic GvHD, and chronic neurological GvHD within 120 days after allo-HSCT, so he had been receiving anti-GvHD treatment intermittently. In November 2021, he attempted to interrupt antiretroviral therapy, and no plasma viral load was detected in the subsequent 32 months.

The emergence of the sixth case defied the common concept that CCR5 Δ32/Δ32 stem cell transplants were needed for HIV cure, making the mechanism of HIV cure through allo-HSCT even more intricate. In this case, the patient suffered from recurrent GVHD, which was even more severe than in earlier cured cases. During the analytical treatment interruption (ATI), ruxolitinib treatment was continuously administered to manage chronic GvHD, with only a short four-week break. Ruxolitinib has been proven to block HIV replication, viral reactivation, and reservoir re-seeding in vitro [83,84], yet no viral rebound occurred in this patient during the interruption of ruxolitinib treatment. High-frequency CD16^+^ CD56^−^ NK cells were detected in the patient’s body after allo-HSCT. Since this parameter has not been examined in other cured cases, it is uncertain what role this cell population plays in the HIV cure process. The researchers reporting this case stated that it is still unknown whether this patient’s disease remission will last indefinitely. There is also the lingering concern that viral rebound might occur once the long-term immunosuppressive drugs are discontinued.

#### 4.1.7. The Second Belin Patient

A 60-year-old German man tested HIV-positive in 2009 and was diagnosed with AML in 2015 [16]. His physicians were unable to find a matching stem cell donor who had mutations in both copies of the CCR5 gene. However, they found a female donor who also had a mutation in one copy (CCR5 Δ32/WT), similar to the patient. The patient underwent allo-HSCT in 2015 and stopped taking ART medication in 2018. Now, nearly six years have passed and his HIV has not rebounded.

The patient was the second person to receive stem cells susceptible to HIV [16] after the “Geneva patient”. If the key factor in these cured cases is that PLWH carrying only one mutated copy of the CCR5 gene receive allo-HSCT with also only one mutated copy, then this finding will expand the donor pool for allo-HSCT because approximately 10% of people of European descent have one mutated copy of the CCR5 gene [85]. The more detailed treatment process of this patient has not been disclosed yet, and it is not possible to conduct a more in-depth comparison between this case and other cured cases for the time being.

#### 4.1.8. The Eighth Cured Case

Recently, a patient who received allo-HSCT was reported at the HIV Glasgow 2024 conference and might become the eighth case of HIV cure worldwide [86]. This patient was diagnosed with HIV infection in 1999 and received ART in the early stage. By 2010, the HIV viral load in her body had become undetectable. She developed AML in February 2020 and underwent splenectomy due to a ruptured subcapsular hematoma two months later. She received CCR5Δ32/Δ32 allo-HSCT from an HLA-mismatched (HLA-A) donor in July 2020 after undergoing a Baltimore-based conditioning regimen and GVHD prophylaxis. In October 2020, she developed acute cutaneous GVHD and recovered soon. ATI occurred in October 2023, which was 39 months after allo-HSCT. There has been no rebound in viral load ten months after the discontinuation of medication, accompanied by a continuous decline in the titer of HIV-1 antibodies. This newly reported case is similar to previous successful cases such as the “Berlin patient” and the “London patient”, and it is a typical case of CCR5 Δ32/Δ32 allo-HSCT cure.

**Table 1:** Summary of cases of HIV cured by allo-HSCT.

Items	Berlin Patient	London Patient	Düsseldorf Patient	New York Patient	City of Hope Patient	Geneva Patient	The second Berlin Patient	The Eighth Cured Case
Basic information								
Gender	Male	Male	Male	Female	Male	Male	Male	Female
Age at transplantation	40	36–37	43	59	63	47	51	50s
Pre-transplant HIV infection status								
Duration of HIV infection (Y)	>12	>13	>4	>4	>31	>28	>6	>21
Duration of ART (Y)	4	NM	2	>4	~21	~28	NM	NM
Cancers information								
Type	AML	HL	AML	AML	AML	Myeloid sarcoma	AML	AML
Treatment	Induction CT + consolidation CT	First-line CT + salvage regimens	Induction CT + consolidation CT	Induction CT + consolidation CT	Salvage regimens	Induction CT/ hypomethylating agent	NM	NM
Allo-HSCT information								
HSC sources	PBSCs	PBSCs	PBSCs	UC/PBSCs	PBSCs	PBSCs	Bone marrow	NM
HLA Matching	10/10	9/10	10/10	5/8 UC + 4/8 PBSCs	11/12	9/10	NM	9/10
CCR5 genotype—donor	Δ32/Δ32	Δ32/Δ32	Δ32/Δ32	Δ32/Δ32	Δ32/Δ32	WT/WT	Δ32/WT	Δ32/Δ32
CCR5 genotype—recipient	Δ32/WT	WT/WT	Δ32/WT	WT/WT	WT/WT	WT/WT	Δ32/WT	NM
Dose of stem cells (per kg body weight)	2.3 × 10^6^ (First) 2.1 × 10^6^ (Second)	3.6 × 10^6^	8.74 × 10^6^	2 × 10^7^ CB cells + 2.2 × 10^5^ PBSCs	NM	NM	NM	NM
HSC transplant times	2	1	1	1	1	1	1	1
Post-transplant donor fusion time (D)	13	30	34	~98	30	<30	NM	NM
Preconditioning for allo-HSCT								
Chemotherapy	High intensity	Reduced intensity	Reduced intensity	High intensity	Reduced intensity	High intensity	NM	Yes
Irradiation	Yes (200 cGy)	No	No	Yes (4 Gray)	No	Yes (8 Gray)	NM	Yes (200 cGy)
GVDH information								
Type and degree	Skin, grade I	Gastric/duodenal/ colonic biopsies, grade I	Eye, mild	No	Mild	Liver, acute/ skin, Mild/nervous system, chronic	NM	Skin, acute
Preventive medications	Rabbit anti-thymocyte globulin/cyclosporine/ mycophenolate mofetil	Anti-CD52/ cyclosporine/ methotrexate	Cyclosporine/ mycophenolate mofetil/ tacrolimus	Mycophenolate mofetil/ tacrolimus	Sirolimus/ tacrolimus prophylaxis	Cyclophosphamide/ Tacrolimus/ mycophenolate	NM	cyclosporin A/ mycophenolate mofetil/ cyclophosphamide
Treatment	Cyclosporine	No	NM	No	NM	Various drugs	NM	NM
ART interruption information								
Time of ATI	1 day before transplantation	16 months after transplantation	69 months after transplantation	37 months after transplantation	51 months after transplantation	40 months after transplantation	3 years after transplantation	39 months after transplantation
Length of HIV remission (ATI initiated)	12 years (2008)	7 years (2017)	6 years (2018)	4 years (2020)	3 years (2021)	3 years (2021)	5 years (2019)	1 years (2023)
References	[10]	[11]	[12]	[13]	[14]	[15]	[16]	[86]

Allo-HSCT: hematopoietic stem cell transplantation; Y: year; D: day; AML: acute myeloid leukemia; HL: Hodgkin lymphoma; CT: chemotherapy; IR: irradiation; PBSCs: peripheral blood stem cells; CB: cord blood; NM: not mentioned; and ATI: analytical treatment interruption.

### 4.2. Potential Factors for Sterlizing Cure

#### 4.2.1. Hematopoietic Stem Cells with CCR5 Δ32/Δ32 Mutation

The viral envelope trimer complex composed of the heterodimeric proteins gp120 and gp41 is essential for HIV to recognize and enter target cells [87,88]. When gp120 binds to the CD4 protein, structural changes occur in the viral envelope complex, exposing specific domains in gp120 that can bind to chemokine receptors on the cell membrane [89,90]. Up to now, at least 17 members of such chemokine ligands have been identified as co-receptors for HIV. The most common co-receptors for HIV are CXCR4 (C-X-C chemokine receptor type 4 or CD184) and CCR5 (C-C chemokine receptor type 5) [91]. CXCR4 is expressed on a variety of cells including T lymphocytes, while CCR5 is present on monocytes/macrophages, dendritic cells, and activated T lymphocytes [92,93,94]. HIV strains that use CCR5 to enter cells are called R5-tropic, those that use CXCR4 are called X4-tropic, and strains that can use both are called R5X4 or dual-tropic [94,95]. As mentioned before, five of the seven cured cases described in this article received allo-HSCT carrying a homozygous mutation in the CCR5 gene (CCR5Δ32/Δ32). Cells carrying this mutation lack CCR5 on their surface and have a natural resistance to R5-tropic HIV strains. The HIV carried by these five individuals before being cured was mainly R5-tropic, and some individuals carried a very small amount of X4-tropic virus strains. All of them achieved long-term remission after receiving allo-HSCT carrying CCR5Δ32/Δ32, suggesting that the deletion of the CCR5 gene on stem cells is an important factor in the cure of HIV. Intervening at the CCR5 target is expected to break the dilemma of the continuous replication and difficulty in clearing HIV in the body, opening up a highly promising new path for the HIV cure [96,97].

It should be noted that not all PLWH who received allo-HSCT with CCR5Δ32/Δ32 have been cured. A 27-year-old patient with HIV infection and anaplastic large cell lymphoma had his HIV strain’s tropism shift from mainly R5 to X4 after receiving CCR5Δ32/Δ32 stem cell transplantation, which led to viral rebound after the interruption of ART [98]. The patient discontinued ART before the start of myeloablative treatment but resumed it three weeks after transplantation due to the rebound of HIV. Nearly one year after transplantation, ART was interrupted because of the recurrence of the patient’s T cell lymphoma, and the virus rebounded rapidly about one month after the interruption. This failure case indicates that the cure of HIV does not solely depend on the mutation status of the CCR5 gene on stem cells, but also requires the synergistic action of other factors. The deletion of the CCR5 gene on donor cells can prevent the transplanted cells from being infected by HIV and reduce the risk of HIV rebound. However, long-term remission of HIV does not seem to be achievable only through receiving allo-HSCT carrying the homozygous mutation of CCR5, such as the “Geneva patient” and “The second Berlin patient”. An experiment using cynomolgus monkeys to explore the mechanism of HIV cure also proved this point, suggesting that the homozygous mutation of the CCR5 gene may not be a necessary factor for the cure of HIV [99].

#### 4.2.2. Allogeneic Immunity

Allo-HSCT can trigger two types of immune responses. One is that donor-derived CD4^+^ T cells replace recipient CD4^+^ T cells, and the other is that the donor’s immune cells attack the recipient’s tissues and organs (GVHD) [99]. The combined action of these two responses can effectively clear HIV latent cells and reduce the HIV reservoir [100]. Allogeneic immunity mediates the graft-versus-reservoir (GVR) effect in HIV infection and is considered to be one of the contributing factors for the elimination of HIV-infected cells in the cured cases of HIV, including the “Berlin patient”, “London patient”, “Düsseldorf patient”, and especially the “Geneva patient”. Studies on Mauritian cynomolgus macaques (MCM) have shown a statistically significant negative correlation between the donor chimerism rate and SIV DNA. This correlation links allogeneic immunity to reservoir clearance [74]. This study also found that in SIV-infected MCM, those receiving CCR5 wild-type allo-HSCT cleared the viral reservoir when they had GVHD. In contrast, those without GVHD experienced a viral rebound. This further demonstrates that allogeneic immunity is the main mechanism for clearing the latent viral reservoir after allo-HSCT [74,101].

The allogeneic response partly explains why the “Geneva patient”, who only received allo-HSCT with CCR5 wild-type, successfully cleared HIV, as this patient had a relatively severe GVHD reaction after transplantation [14]. However, without the protection of transplanted cells provided by the CCR5 deficiency, many patients who only received CCR5 WT/WT allo-HSCT experienced a viral rebound after discontinuing ART, such as “Boston patients” [102]. Thus, unless latent HIV in patients can be completely eradicated, without the protection for transplanted cells offered by CCR5 deficiency, relying merely on GVHD is insufficient. GVHD is also the main cause of death in patients after allo-HSCT [103].

How to utilize allogeneic immunity other than transplantation to obtain new HIV cure methods that can be more widely applied to PLWH is an important direction for future research. The detailed characterization of allo-reactive T cells from transplanted individuals, such as the analysis of T cell receptors, antigen specificity, and target recognition, will provide a wealth of information and may form the basis of new T cell therapies for HIV cure [17].

#### 4.2.3. Pre-Transplant Conditioning

During the process of allo-HSCT, pre-transplant conditioning is used to reduce the burden of malignant cells and create space for the engraftment of donor hematopoietic cells [104]. For patients who are also infected with HIV, pre-transplant conditioning can also partially shrink the HIV reservoir [105], preparing for the donor-derived cells to clear and replace the host’s infected cells. Among the seven cured cases, the preconditioning regimens are divided into two types: one is myeloablative high-intensity conditioning (HIC), and the other is non-myeloablative reduce-intensity conditioning (RIC). As can be seen from the three cases of the “London patient”, “Düsseldorf patient” and “City of Hope patient”, for patients who receive CCR5 Δ32/Δ32 allo-HSCT, RIC will not change the outcome of the patients’ cure [12,14,82]. However, for patients who receive allo-HSCT with wild-type CCR5, such as the “Geneva patient”, RIC may not be sufficient [15]. For these patients, the transplanted cells could easily infected by HIV, so every possible means must be taken to minimize the HIV reservoir before transplantation. Also, undergoing CCR5 WT/WT allo-HSCT, the “Geneva patient” adopted a myeloablative HIC regimen during the treatment process and finally achieved clinical cure successfully, while the “Boston patients” only received RIC, and allo-HSCT did not bring them the same desirable treatment effect [15,102]. The comparison between the two highlights the importance of myeloablative high-intensity preconditioning for patients who receive allo-HSCT with wild-type CCR5. Therefore, accurately determining the breakpoint for making a choice between RIC and HIC is undoubtedly a vitally important issue. When choosing the pre-transplantation conditioning regimen, it is essential to comprehensively consider various factors, such as the patient’s age, physical condition, cancer burden, viral load, viral tropism, and the CCR5 gene mutation status of the donor and the recipient.

Although HIC contributes to allo-HSCT-mediated HIV cure, even under the strict maintenance of ART, myeloablative HIC is insufficient to eradicate the HIV reservoir. For the “Berlin patient” who received HIC, HIV DNA could be easily measured in his peripheral blood samples after transplantation, indicating that the preconditioning regimen alone cannot achieve the cure of HIV [10]. Studies using macaques for whole-body imaging have shown that splenic CD4^+^ cells are resistant to the depletion caused by myeloablative total body irradiation (TBI) or anti-CD3 immunotoxin, which may explain why the preconditioning regimen cannot eradicate all HIV-infected cells [106]. It should be noted that myeloablative regimens such as TBI have a strong immunosuppressive effect and pose an overly high risk when used in PLWH without fatal malignancies. Therefore, it is particularly important to clarify the depletion capabilities of various conditioning regimens on the viral reservoir and identify safe regimens applicable to HIV patients receiving stable ART. During this process, a careful balance needs to be struck between the benefits of HIV cure and the significant risks of the conditioning regimens.

#### 4.2.4. ART

ART is also an important auxiliary factor in the process of HIV cure mediated by allo-HSCT. The seven cured patients all received ART treatment before transplantation and kept the viral load at a very low level. To protect the transplanted cells from HIV infection, except for the “Berlin patient”, the other cured cases maintained ART treatment for at least one year after transplantation. In particular, for the cured cases in which stem cells susceptible to HIV were transplanted, such as the “Geneva patient” and “The second Berlin patient”, ART treatment was even maintained for three years after transplantation to reduce the risk of viral rebound [15,16]. A patient who received stem cell transplantation (CCR5 Δ32/Δ32) stopped ART before the start of myeloablative treatment and experienced viral rebound three weeks after transplantation; until two weeks before death, the patient still had a high viral load in the body [84]. Previous case reports and prospective studies have shown that donor cells are prone to infection before achieving full chimerism [75,107]. Therefore, the continuation of fully suppressive ART is essential before the graft achieves full chimerism. However, a study using MCM showed that despite receiving ART, the virus could still spread to donor-derived cells in the early stage after transplantation [74]. This phenomenon may be related to the fact that ART drugs cannot fully penetrate all tissues and fully inhibit HIV replication [108,109]. So, in the future, it is still necessary to explore more efficient treatment methods to prevent grafts from being infected by HIV.

In summary, the sterilizing cure of HIV mediated by allo-HSCT is facilitated by multiple factors, mainly including the following: ① CCR5 homozygous mutant stem cells; ② allogeneic immunity; ③ pre-transplant conditioning; and ④ fully suppressive ART. These factors constitute the core mechanism for the cure of HIV. That is, first, the level of HIV is controlled by ART. Then, the HIV reservoir is reduced through chemotherapy and radiotherapy. Finally, the remaining infected cells are replaced and cleared through allo-HSCT. Coupled with the natural barrier created by the absence of CCR5 on donor cells, the possibility of HIV rebound is eliminated. However, it must be noted that patient-specific factors, such as genetic and immunological ones, likely play a role in the success of this curative approach. Although this approach has achieved remarkable success in a few cases, it is not applicable to all people infected with HIV. The reason is that allo-HSCT is a treatment method with extremely high risks, involving high-dose radiotherapy and chemotherapy, and may produce severe side effects such as GVHD [110]. In addition, finding suitable donors is a complicated process, and more research is still needed to explore how to extend this curative strategy to a broader patient population [85].

## 5. Possible Future Strategies for Functional Cures of HIV

### 5.1. Transcriptional Regulation of Latent Viruses

#### 5.1.1. Shock and Kill

**Figure 1:**
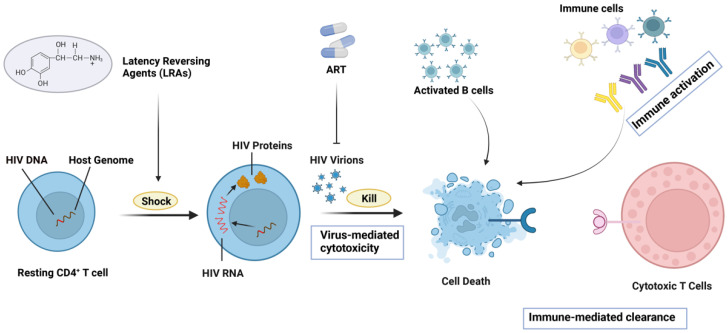
The “Shock and kill” strategy for treating HIV. This figure was created with BioRender.com.

The “shock and kill” strategy intends to employ latency-reversing agents (LRAs) to trigger latent virus transcription and then “eliminate” these reservoir cells via cytopathic effects, host immune responses, or other targeted means (Figure 1) [111]. Currently, studied LRAs are grouped into two types. One is epigenetic modifiers, such as histone deacetylase inhibitors (HDACis), histone methyltransferase inhibitors (HMTis), bromodomain and extraterminal domain inhibitors (BETis), and DNA methyltransferase inhibitors (DNMTis), etc. All of these substances can reverse the repressive epigenetic marks present on the HIV promoter during latency [112,113,114,115]. The other type consists of activators of inducible host factors, including Toll-like receptor (TLR) agonists, activators of canonical NF-κB signal transduction, and second mitochondrial-derived caspase (Smac) mimetic activators [116,117,118]. This type of LRA forces infected cells to enter an activated state by activating key signaling pathways and promoting the transcription of viral genes [119]. Once latently infected cells are activated and start producing the virus, the immune system can more readily identify and wipe them out [120]. At this time, antiretroviral therapy (ART) and immunotherapy can both reduce the risk of viral replication after activation of latently infected cells and enhance the attacking power of the immune system against infected cells. Although this treatment strategy is theoretically feasible, existing LRAs have not delivered satisfying results in HIV treatment [121]. Indeed, LRAs can induce HIV transcription in vitro and in animal models but fail to significantly reduce latent viral reservoir size in clinical trials [122]. This failure of LRAs may be explained by limited reactivation of the HIV provirus and/or of antigen expression, mutations in epitopes impairing immune recognition, ineffective magnitude or functionality of immune effectors, or intrinsic resistance of cells to elimination [123]. Moreover, many LRAs activating proviral expression also spark wide immune activation or severe side effects, unfit for clinical use [124]. To boost this treatment, we need to dig deeper into the latent HIV transcription mechanism to develop more targeted, potent, and safe LRAs.

#### 5.1.2. Block and Lock

“Block and lock” is a strategy designed to permanently suppress the viral transcription mechanism, aiming for a functional cure of HIV [125,126,127]. It uses latency-promoting agents (LPAs) to “block” viral transcription, leading the latent viral reservoir into a state of permanent silence, known as “super latency” or “deep latency” [128]. Based on the blocking mechanism, LPAs fall into two categories. One category induces viral silencing via RNA, like short interfering (si)RNA, short hairpin (sh)RNA, and long non-coding RNA (lncRNA) [129,130]. The other category blocks the recruitment of RNA polymerase II and histone acetyltransferase (HAT) by inhibiting the Tat protein, such as didehydro–cortistatin A (dCA) and Nullbasic [131,132]. This method has the advantage of skipping complex activation and clearance, potentially offering a more lasting solution [133]. But assessing any “block and lock” protocol’s efficacy demands considering three points: ① whether it targets and reaches all latently infected cells; ② if there are off-target effects; and ③ how long the induced viral silencing lasts. Given these uncertainties, implementing this strategy requires continuous monitoring of viral load and immune status to keep the virus “locked” [121]. Unlike many drugs repurposed for “activate and kill” therapies, those for “block and lock” are new molecules, which are not yet FDA-approved. Hence, clinical trials for related drugs are progressing slowly [134].

### 5.2. Gene Editing

**Figure 2:**
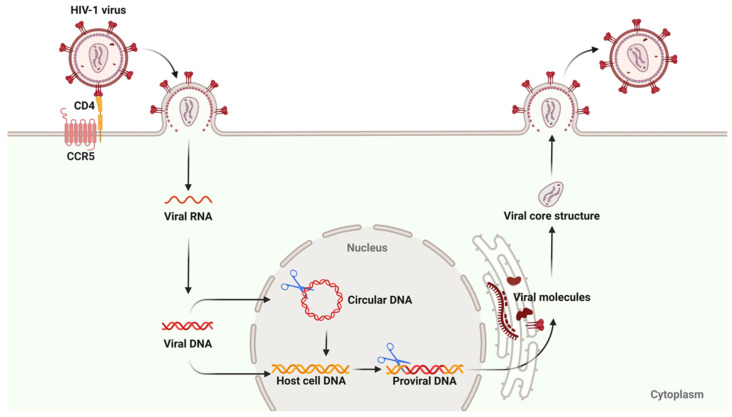
The potential HIV target sites using gene editing. This figure was created with BioRender.com.

Gene-editing technologies, such as CRISPR-Cas9, TALENs, and ZFN (Zinc Finger Nucleases), have shown great potential in HIV cure research [135,136]. These technologies prevent viral invasion and replication by targeting and cleaving HIV DNA or modifying the CCR5 gene in host cells (Figure 2). CRISPR-Cas9, in particular, has been the focus of research due to its high efficiency and specificity. Recent studies have demonstrated that CRISPR-Cas9 can effectively excise a part of the HIV genome in vitro and in vivo experiments, thereby preventing viral replication [25]. In addition, immune cells resistant to R5-tropic HIV can be generated by modifying stem cells through gene editing techniques so that they are absent of the CCR5 receptor. These modified stem cells proliferate in the patient and replace infected cells, thus providing long-term immune protection [137,138]. It is worth noting that in the case of disrupting the CCR5 co-receptor, patients remain vulnerable to infection by X4-tropic viral strains. One benefit of these gene editing sequence approaches is the high specificity required to match the target sequence. However, this also means that due to the extreme sequence diversity within the HIV genome, combinations of multiple sequences will be required to ensure sequence diversity and address the possibility of future viral mutations at the target locus. This raises the complexity and cost of gene editing therapies, obstructing the extensive application of such therapies [139]. In addition, there have been relevant reports on viral escape caused by low gene editing efficiency and off-target effects [21,140].

### 5.3. Immunotherapy

#### 5.3.1. Vaccines

The goal of HIV vaccines is not only to prevent infection but more importantly to help PLWH achieve long-term control and functional cure of the virus [141,142]. The development of preventive vaccines is fraught with challenges. The RV144 trial in Thailand is currently the largest-scale HIV vaccine trial. However, it only shows a 31% protective efficacy in preventing viral infections, and its effect is almost negligible [143,144] Research on therapeutic vaccines aims to boost the virus-killing ability of the immune systems of PLWH. Although many of these therapeutic HIV vaccines have led to improvements in autologous HIV-specific T cell responses, their impact on viral control (defined as a delay in viral rebound time or a reduction in the viral load set-point after stopping ART) is generally limited [145,146]. In recent years, research on T cell vaccines has become a hot topic [147]. Researchers are looking at using T cells in PLWH to recognize and attack infected cells. Novel vaccines using mRNA technology, such as the HIV vaccines developed by Moderna and BioNTech, have entered clinical trials [148]. However, due to the lack of an ideal animal model, it is still difficult to directly extend the application of some vaccines that have shown effectiveness in mouse models or primate models to humans, which has significantly slowed down the research progress of HIV vaccines [149,150]. The biggest problem in the development of HIV vaccines is the great variety of HIV strains. To create a vaccine with broad efficacy, it is necessary to find an immunogen that can trigger a protective response against most strains [151].

#### 5.3.2. Neutralizing Antibodies

Broadly neutralizing antibodies (bNAbs) are the focus of HIV-neutralizing antibody research. bNAbs recognize and neutralize a wide range of HIV strains and have broad-spectrum antiviral activity [152,153]. In recent years, several bNAbs have shown encouraging results in clinical trials. For example, VRC01, a broadly neutralizing antibody, has been demonstrated to significantly reduce viral load in treatment-naïve PLWH [154,155]. Another key study was a combination therapy using bNAbs. Studies have shown that a single bNAb may not be sufficient to consistently suppress HIV because the virus can rapidly produce mutations [156]. Therefore, researchers have developed combination therapies with multiple bNAbs, such as the combination of 3BNC117 and 10-1074. These combination therapies have demonstrated significant antiviral efficacy in clinical trials, enabling more effective viral control and reducing the risk of viral rebound [156,157]. In addition to traditional injection therapy for bNAbs, gene therapy techniques have also been used in bNAb research. Delivery of the bNAb gene using adeno-associated virus (AAV) vectors provides sustained viral control by allowing long-term expression of neutralizing antibodies in patients. Early clinical trials have demonstrated the safety and potential efficacy of AAV vectors for bNAbs, providing a new direction for long-term antibody therapy [158,159]. In addition, researchers are exploring the use of bNAbs in combination with other therapeutic approaches, such as ART and immunomodulators, to achieve more comprehensive viral control and immune restoration. Nevertheless, due to issues such as the diversity of HIV, the latent viral reservoir, and the degradation of bNAbs, the efficacy of bNAbs remains rather limited [160]. Moreover, the exorbitant production costs and the administration method via blood transfusion have restricted the clinical application of bNAbs [161]. How to reduce viral drug resistance and develop bNAbs that are more broadly effective and have a long half-life is an important direction for future research in this field [162].

#### 5.3.3. Nanobodies

Nanobodies have potential applications in HIV diagnosis, vaccine design, microbicides, immunoprophylaxis, and immunotherapy due to their small molecular mass, high tissue penetration ability, high affinity, and low immunogenicity [163]. They can be attached to the human Fc chain for immune effector function, and recognition of bivalent and trivalent nanobodies with the same or different epitopes on the envelope glycoproteins gp120 and gp41 greatly increases the potency of HIV neutralization [164]. Nanosomes 238D2 and 238D4 bind cytosolic CXCR4 and neutralize the X4 strain of HIV by blocking this co-receptor [165], and intracellular nanosomes (endosomes) interfere with viral production through Rev inhibition of multimerization [166]. Anti-p24 nanosomes have been used as detectors of p24 Gag antigen for the design of miniaturized diagnostic tests for HIV infection [167,168]. Meanwhile, several groups isolated nanobodies targeting the intracellular HIV proteins, Rev, Nef, and Vpr, which are essential for HIV replication in human cells. Vercruyse et al. chose nanobodies interfering with the assembly of Rev polyprotein complexes for viral RNA processing and export [166]. Bouchet et al. showed that intracellular expression of Nef-specific nanobodies blocked the HIV replication cycle [169]. Matz et al. skillfully utilized a yeast two-hybrid approach to select a single-domain antibody that binds to Vpr and prevents its translocation from the cytoplasm to the nucleus [170]. Nanobodies, as an emerging therapeutic tool with good specificity and low side effects, still need to be improved in terms of stability and broad spectrum to cope with viral mutations.

### 5.4. Chimeric Antigen Receptor Therapy

**Figure 3:**
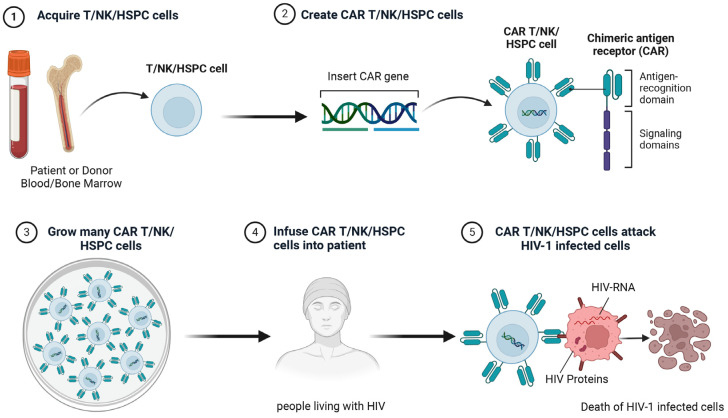
CAR-T, CAR-NK, or CAR-HSPC therapy for HIV infection. This figure was created with BioRender.com.

Chimeric antigen receptor (CAR) therapy is a cutting-edge immunotherapy approach [171]. By means of genetic engineering techniques, an artificial receptor known as the chimeric antigen receptor is fabricated [172]. Essentially, this receptor links the variable region of an antibody capable of identifying specific antigens to the activation signal transduction domain of immune cells, facilitating the targeted assault of immune cells on the intended diseased cells (Figure 3) [173]. HIV can infect multiple types of cells, and the latently infected cells hardly express complete viral proteins [174]. How to find specific targets that can target all infected cells while ensuring safety is the biggest problem that CAR therapy faces in the process of HIV treatment.

#### 5.4.1. CAR-T

CAR-modified T cells specifically recognize and kill cells expressing HIV antigens, which may be a promising strategy for the treatment of HIV. In addition, CTL mediates lysis of HIV-infected cells via MCH-I molecules; however, HIV-negative regulatory factor (Nef) has been shown to down-regulate the surface expression of MHC-I molecules in infected cells to evade such immune responses [175]. CAR-T cells can directly recognize antigens in the absence of MHC-I restriction and therefore overcome this viral escape mechanism. Furthermore, given the functional heterogeneity of HIV-specific CD4^+^ T cells and the ability to orchestrate antiviral immunity, therapeutic interventions to restore or enhance CD4^+^ T cell function may be critical for the development of effective HIV cure strategies. A recent study demonstrated that CD4^+^ T cells expressing HIV-specific CARs can directly control HIV replication and enhance virus-specific CD8^+^ T cell responses, highlighting the therapeutic potential of engineered CD4^+^ T cells as a functional HIV cure [176]. Furthermore, studies have demonstrated the antiviral activity of CAR-T cells against HIV reservoirs [177,178]. Thus, CAR-modified T cells may have the advantage of being actively transported to tissue reservoirs (e.g., lymph nodes and rectal tissues), offering new possibilities for reducing HIV reservoirs. Clinical trials have verified the safety of CAR-T therapy. Nevertheless, its major limitations lie in the absence of dose control and a mechanism capable of triggering the activation and proliferation of T cells to offer continuous protection [179,180].

#### 5.4.2. CAR-NK

In addition to CAR-T cells, therapies based on CAR-NK cells have emerged as a promising approach for eradicating HIV-infected cells with antigen-recognition capabilities [181]. Numerous studies have shown that in addition to T cells, NK cells play an important role in fighting HIV infection [182,183]. Notably, Mavilio et al. reported results indicating that in viremic PLWH, CD56^−^ NK cells were significantly expanded, secreted fewer cytokines associated with the initiation of antiviral immune response, and had severely impaired cytotoxicity in this subset compared with CD56^+^ NK cells, which may be related to continued viral replication [184]. In addition, uncontrolled viral replication in HIV controllers is associated with a reduced NK cell response [185]. CAR-NK cells have several advantages over CAR-T cells, including the absence of the need for HLA compliance, thus reducing the risk of graft-versus-host disease, reducing the risk of cytokine release syndrome, and conferring a tumor-limiting effect [186]. Considering the advantages of CAR-NK cell therapy and the role of NK cells in HIV infection, there is great interest in developing strategies for CAR-NK cell therapy for HIV. However, NK cell-based therapies face multiple challenges: MHC class I chain-related protein A shedding in chronic HIV infection [187]; immune cell exhaustion due to long-term antigen stimulation; reduced NK cell function after cryopreservation and transport; weak cytotoxicity of NK cells from chronically infected patients; lack of efficient gene transfer methods; and high cost and long preparation time for CAR-modified immune cells [188]. Future research must address these issues to optimize HIV treatment strategies using NK cells.

#### 5.4.3. CAR-Hematopoietic Stem and Progenitor Cells (CAR-HSPCs)

Allo-HSCT is the only known treatment that can completely cure HIV [189]. In addition to hematopoietic stem and progenitor cell (HSPC)-based gene therapy, CAR-HSPC therapy has emerged as a powerful immunotherapeutic treatment for HIV infection, with demonstrated success in long-term implantation and generation of anti-HIV CAR cells [190]. Modifying HSPCs with HIV-specific CD4 CARs allows them to differentiate into HIV-specific T cells and other cells that may migrate to multiple anatomical sites and inhibit HIV replication in vivo [191]. Another study also showed that HSPC-derived cells could differentiate into functional T cells or NK cells in humanized mice, thus inhibiting HIV replication [192]. Zhen et al. reported the development of a second-generation CD4-based anti-HIV CAR based on hematopoietic stem cells. They noted that this superior CAR exhibited better HSC differentiation and HIV inhibition as well as fewer deleterious functions than the CD4 CAR [192]. These results strongly suggest that CAR-HSPC therapy may be feasible and effective in eliminating the HIV reservoir and promoting a functional cure for HIV infection.

## 6. Clinical Research of Cell Therapy Towards HIV Cure

With regard to clinical research, a number of therapeutic approaches to experimental cell therapy, such as modified CAR-T cell therapy and HIV-specific T cell therapy, are also underway. These therapeutic strategies aim to boost the host immune response and enhance the control of HIV, thereby reducing or eliminating the need for long-term ART. Table 2 lists a number of relevant clinical studies that are ongoing or completed both nationally and internationally [193,194,195].

## 7. Conclusions and Perspectives

In this review, we summarize current research advances in the functional cure of HIV, with a special focus on cases of HIV cured by allo-HSCT, and analyze the potential mechanisms and limitations of this approach. In addition, we also explore feasible strategies for future functional HIV cures, including trends in emerging fields, such as gene editing and immunotherapy, and highlight challenges facing current research, such as the difficulty of removing reservoirs, and the safety and feasibility of clinical treatments. However, with the advancement of science and technology and the continuous depth of research, we are optimistic about realizing a functional cure for HIV. Future research should focus on the development of novel therapeutic strategies and clinical trials while keeping a close eye on potential side effects and safety issues. Overall, this review provides important references and insights for future research and clinical practice and points to the future direction for realizing a functional cure for HIV.

## Figures and Tables

**Table 2:** Clinical trials of cell therapy for HIV treatment.

Cell Therapy Clinical Trials	Trail Registry Identifiers	Sponsors	Phase	Estimated End Date
CAR-T Cells for HIV Infection	NCT04648046	Steven Deeks	Phase I/II	2028-12-31
CMV-specific HIV-CAR T Cells as Immunotherapy for HIV/AIDS	NCT06252402	City of Hope Medical Center	Early Phase I	2028-03-30
Immune Cell Therapy (CAR-T) for the Treatment of Patients With HIV and B-Cell Non-Hodgkin Lymphoma	NCT05077527	AIDS Malignancy Consortium	Phase I	2027-01-31
Safety and Survival of Genetically Modified White Blood Cells in HIV-infected Twins: The Gemini Study	NCT04799483	National Institute of Allergy and Infectious Diseases (NIAID)	Not listed	2030-01-30
CD4^+^ CAR ZFN-modified T Cells in HIV Therapy	NCT03617198	University of Pennsylvania	Phase I	2027-12
HIV-1-Specific T Cells (HST-NEETs) for HIV-Infected Individuals	NCT03485963	Catherine Bollard	Phase I	2024-12
Study of Anti-HIV Cellular Therapy Based on Dendritic Cells Pulsed With Chemically Inactivated Virus	NCT02766049	University of Sao Paulo General Hospital	Phase I/II	2014-05
Lymphocyte Infusions for the Treatment of HIV-Infected Patients Failing Anti-HIV Therapy	NCT00559416	National Institutes of Health Clinical Center (CC)	Phase I	2016-09-30
Redirected High-Affinity Gag-Specific Autologous T Cells for HIV Gene Therapy	NCT00991224	University of Pennsylvania	Phase I	2014-01
Adoptive Transfer of Haploidentical NK Cells and N-803	NCT03899480	University of Minnesota	Phase I	2021-04-01
Phase I Study of HIV-1 Antigen Expanded Specific T Cell Therapy	NCT02208167	University of North Carolina, Chapel Hill	Phase I	2017-11-27
Safety and Effectiveness of Immunotherapy With Autologous HIV-Specific CD8 Cells in HIV-Infected Adults	NCT00110578	National Institute of Allergy and Infectious Diseases (NIAID)	Phase I	2005-04
Effect of Chidamide Combined With CAT-T or TCR-T Cell Therapy on HIV-1 Latent Reservoir	NCT03980691	Guangzhou 8th People’s Hospital	Phase I	2020-05-31
Evaluate the Tolerability and Therapeutic Effects of Repeated Doses of Autologous T Cells With VRX496 in HIV	NCT00295477	University of Pennsylvania	Phase I	2013-12
An Efficacy and Safety Study of shRNA-modified CD34^+^ Cells in HIV-infected Patients	NCT03517631	Shanghai Public Health Clinical Center	Phase I	2020-12-31
Redirected MazF-CD4 Autologous T Cells for HIV Gene Therapy	NCT01787994	University of Pennsylvania	Phase I	2017-07
Safety and Survival of Genetically Modified White Blood Cells in HIV-Infected Persons—A Study in Identical Twin Pairs	NCT00001353	National Institute of Allergy and Infectious Diseases (NIAID)	Phase I/II	2002-03
A Study of Cytotoxic T Lymphocyte (CTL) Therapy in HIV-Infected Patients	NCT00000824	National Institute of Allergy and Infectious Diseases (NIAID)	Not listed	2005-06

## Data Availability

Not applicable.
